# Advancing Photoelectrochemical Energy Conversion through Atomic Design of Catalysts

**DOI:** 10.1002/advs.202104363

**Published:** 2021-12-01

**Authors:** Erling Zhao, Kun Du, Peng‐Fei Yin, Jingrun Ran, Jing Mao, Tao Ling, Shi‐Zhang Qiao

**Affiliations:** ^1^ Key Laboratory for Advanced Ceramics and Machining Technology of Ministry of Education Tianjin Key Laboratory of Composite and Functional Materials School of Materials Science and Engineering Tianjin University Tianjin 300072 China; ^2^ School of Chemical Engineering and Advanced Materials The University of Adelaide Adelaide SA 5005 Australia

**Keywords:** atomic design, photoelectrochemical ammonia generation, photoelectrochemical carbon dioxide reduction, photoelectrochemical hydrogen evolution

## Abstract

Powered by inexhaustible solar energy, photoelectrochemical (PEC) hydrogen/ammonia production and reduction of carbon dioxide to high added‐value chemicals in eco‐friendly and mild conditions provide a highly attractive solution to carbon neutrality. Recently, substantial advances have been achieved in PEC systems by improving light absorption and charge separation/transfer in PEC devices. However, less attention is given to the atomic design of photoelectrocatalysts to facilitate the final catalytic reactions occurring at photoelectrode surface, which largely limits the overall photo‐to‐energy conversion of PEC system. Fundamental catalytic mechanisms and recent progress in atomic design of PEC materials are comprehensively reviewed by engineering of defect, dopant, facet, strain, and single atom to enhance the activity and selectivity. Finally, the emerging challenges and research directions in design of PEC systems for future photo‐to‐energy conversions are proposed.

## Graphical abstract

Photoelectrochemical hydrogen/ammonia generation and carbon dioxide reduction offer more opportunities for carbon neutrality. In this review, recent progress in the atomic design of PEC materials for enhanced surficial catalysis is reviewed from the aspects of defect, dopant, facet, strain, and single atom engineering. Emerging challenges and research directions for the design of future photo‐to‐energy devices are also proposed.

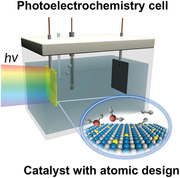

## 1 Introduction

Since industrialization, the global surface temperature has been rising, seriously impacting on the global ecosystem and social and economic environment.^[^ ^1^, ^2^ ^]^ Extreme weather such as tornadoes, rainstorms, heatwaves, and droughts occur frequently.^[^ ^3^, ^4^, ^5^ ^]^ This threatens the stability of human society and hinders the sustainable development of human beings.^[^ ^6^ ^]^ According to the Paris Agreement,^[^ ^7^ ^]^ by the end of the 21st century, the rise in global average temperature must be limited to less than 2 °C above preindustrial levels. At present, more than 50 countries have announced that they will achieve carbon neutrality by the middle of this century, and nearly 100 countries are working on setting their own goals. Carbon neutrality has become a global scale movement.

Until now, fossil energy source, such as coal and oil, is still the main consumption force in the energy structure of all countries. In 2019, fossil energy accounted for 85.1% of the primary energy consumption in China, 83.3% in the United States, 74.1% in the European Union, and 87.4% in Japan.^[^ ^8^ ^]^ The extensive use of fossil energy has led to excessive carbon emissions. Moreover, energy‐intensive industries are also the main source of carbon emissions. For example, the Haber–Bosch process for ammonia (NH_3_) preparation (operating at 400–500 °C, 100–200 atm) accounts for ≈1.5% of global greenhouse gas emissions.^[^ ^9^, ^10^, ^11^ ^]^ To achieve carbon neutrality, it is imperative to develop clean and renewable energy and upgrade the energy‐intensive industry to carbon‐free processes. In addition, the development of carbon capture and storage technology is also an important route to reduce carbon emissions.^[^ ^12^, ^13^ ^]^

**Figure 1:**
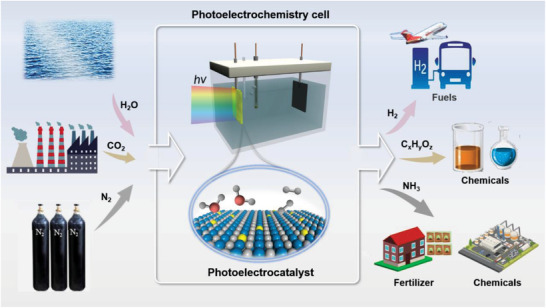
Schematic illustration of PEC energy conversion for the production of H_2_, NH_3_ and the reduction of CO_2_ to valuable chemicals.

Solar energy is an exhaustless natural resource, with 89, 300 TW of solar energy available on the earth's surface, exceeding the world's annual energy consumption.^[^ ^14^, ^15^, ^16^, ^17^ ^]^ Harvesting energy directly from sunlight provides a desirable approach toward fulfilling the requirement for clean and renewable energy. Since the pioneering work of Fujishima and Honda in 1972,^[^ ^18^ ^]^ which reported a photoelectrochemical (PEC) system with TiO_2_ as an electrode to split water, photoelectrochemistry has been considered as a leading environmentally friendly approach for solar to energy (fuel and chemical) conversion. Until now, significant advances have been achieved in PEC hydrogen (H_2_) evolution.^[^ ^19^, ^20^ ^]^ Recently, promising results have also been shown in PEC‐driven NH_3_ generation and carbon dioxide (CO_2_) reduction to valuable chemicals.^[^ ^21^, ^22^ ^]^ Undoubtedly, PEC‐driven production of H_2_, NH_3_ and reduction of CO_2_ under eco‐friendly and mild conditions provide a highly attractive solution to carbon neutrality (**Figure** **1**).^[^ ^23^, ^24^, ^25^, ^26^ ^]^

However, the innovation of the PEC devices with high activity, durability, and selectivity for water‐to‐H_2_, nitrogen‐to‐NH_3_, and CO_2_‐to‐chemicals is highly challenging. The common photoelectrodes are composed of light‐absorbing materials decorated with catalytically active cocatalysts.^[^ ^27^, ^28^ ^]^ The efficiency of solar to fuel/chemical conversion depends on the efficiencies of photocarrier generation,^[^ ^29^, ^30^ ^]^ separation,^[^ ^31^, ^32^ ^]^ transport,^[^ ^33^ ^]^ and the final catalytic reaction.^[^ ^34^ ^]^ The loss of photogenerated carriers in any of these processes mentioned above will affect the final solar‐to‐fuel/chemical conversion efficiency and restrict the further performance enhancement of the PEC system. Technoeconomic modeling has suggested that a practical water splitting device should be able to produce a solar‐to‐hydrogen (STH) efficiency of >10% to compete with the conventional industrial processes (methane steam reforming).^[^ ^20^ ^]^ So far, this requirement has been met through the construction of two series polymer electrolyte membrane electrolyzer driven by InGaP/GaAs/GaInNAsSb triple junction solar cells, and the overall STH efficiency can reach more than 30%.^[^ ^35^ ^]^ By contrast, PEC‐driven nitrogen reduction reaction (PEC‐NRR) and CO_2_ reduction reaction (PEC‐CRR) are still in their infancy, whose performance is far from requirements for wide application.

Recently, thanks to the exciting progress in novel synthetic method,^[^ ^36^, ^37^ ^]^ advanced‐theoretical computations,^[^ ^38^, ^39^ ^]^ and cutting‐edge characterization techniques,^[^ ^40^ ^]^ tremendous breakthroughs have been achieved in nanostructured electrocatalysts toward hydrogen evolution reaction (HER),^[^ ^41^, ^42^ ^]^ NRR,^[^ ^43^, ^44^ ^]^ and CRR.^[^ ^45^, ^46^ ^]^ The rationally designed electrocatalysts with tunable atomic^[^ ^47^ ^]^ and electronic structure^[^ ^48^, ^49^ ^]^ have been obtained. The performance of many cost‐effective and earth‐abundant electrocatalysts has surpassed those of noble‐metal‐based electrocatalysts.^[^ ^50^ ^]^ Until now, special emphasis in PEC system is on the improvement of light‐absorbing, charge separation, and transfer processes,^[^ ^51^, ^52^, ^53^ ^]^ but much less attention is given to the surface catalytic process on the photoelectrocatalysts. Since the catalytic reaction is mainly determined by the electronic structure of the surface atoms,^[^ ^49^ ^]^ the atomic design of the photoelectrocatalysts is urgently needed.^[^ ^54^ ^]^ Besides, appropriate atomic design can bring additional beneficial effects,^[^ ^55^ ^]^ such as extending the light absorption range by introducing defects,^[^ ^56^ ^]^ accelerating charge separation by surface faceting, etc. However, till now, there is no comprehensive review pinpointing the significance of PEC‐HER, PEC‐NRR, and PEC‐CRR with advancements through the atomic design of PEC photoelectrocatalysts.

In this review, we focus on recent progress in the atomic design of photoelectrocatalysts to enhance PEC performance. We focus on the three key reactions for renewable energy and chemical production: HER, NRR, and CRR. For each reaction, the mechanism is discussed briefly. Our intention is to show how atomic design, such as engineering of defect, heteroatom, facet, strain, and single atoms, successfully modulates the electronic structure of photoelectrocatalysts, thus leading to excellent PEC performance. Finally, we provide some insights into the development of photoelectrocatalysts and summarize the remaining challenges and future directions in this emerging field. We hope that this review will offer helpful guidance for oriented design and optimization of photoelectrocatalysts for the research and development of highly efficient PEC systems.

## 2 Reaction Mechanisms of PEC System

**Figure 2:**
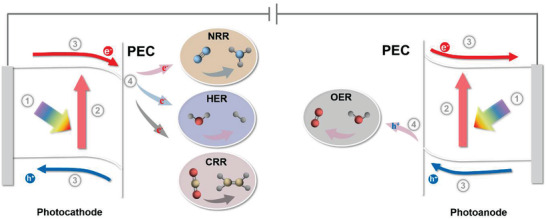
Schematic diagram of four processes of photoinduced charge generation, separation, transfer, and final catalytic reactions in PEC devices.

PEC‐driven energy conversion processes are highly complex because photoinduced charge undergoes generation,^[^ ^57^ ^]^ separation,^[^ ^58^ ^]^ and transfer process^[^ ^59^ ^]^ before reaching the surface active sites for final catalytic reactions. In order to understand the limitations of PEC performance and develop design principles for better PEC materials, it is essential to investigate the principles and mechanisms involved in the above four processes (**Figure** **2**). Given that many excellent works have given comprehensive review^[^ ^60^, ^61^, ^62^, ^63^ ^]^ on the origin of the charge recombination during generation, separation, and transfer processes, and the corresponding strategies to prevent photogenerated charge loss,^[^ ^64^, ^65^ ^]^ we will briefly introduce the mechanisms of water splitting, NRR, and CRR, which will help understanding how atomic design of photoelectrocatalysts significantly affects the performance of PEC systems. Note that the mechanisms of these catalytic reactions in the PEC system are very similar to those in electrocatalysis,^[^ ^66^, ^67^ ^]^ which have been deeply investigated. In this review, the widely accepted catalytic mechanism for the specific reactions are introduced.

### 2.1 Mechanisms for Water Splitting

**Figure 3:**
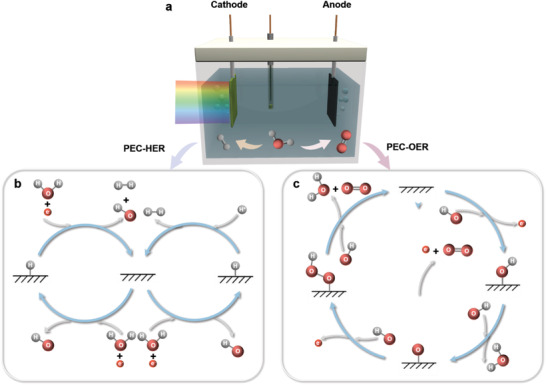
a) Schematic diagram of PEC cell for water splitting under alkaline conditions. b) HER mechanism. c) OER mechanism.

PEC splitting of water into H_2_ and O_2_ is a highly attractive way to produce H_2_ fuel.^[^ ^68^, ^69^, ^70^, ^71^ ^]^ The kinetic and thermodynamic barriers in the water oxidation and reduction reactions are one of the important factors restricting the STH conversion efficiency.^[^ ^72^, ^73^, ^74^ ^]^ In PEC water splitting (**Figure** **3**a), the overall reaction is: 2H_2_O →  2H_2_ + O_2_, which can be conducted in acidic, neutral, and alkaline environments (Figure 3a). We take alkaline water splitting as an example to describe the generally accepted mechanisms.

For water reduction reaction at the photocathode, there are two possible mechanisms (Figure 3b), i.e., Volmer–Tafel and Volmer–Heyrovsky pathways.^[^ ^75^, ^76^ ^]^ For Volmer–Tafel pathway

(1) $$M\;+\;H_{2}O_{\left(l\right)}\;+\;e^{-}\;\to \;M\;-H^{*}\;+\;{\mathrm{OH}}^{-}$$

(2) $$2M\;-\;H^{*}\;\to \;2M\;+\;H_{2}$$

For Volmer–Heyrovsky pathway

(3) $$M\;+\;H_{2}O_{\left(l\right)}\;+\;e^{-}\;\to \;M\;-{\;H}^{*}+\;OH^{-}$$

(4) $$M\;-\;*H\;+\;H_{2}O_{\left(l\right)}\;+\;e^{-}\;\to \;M\;+\;OH^{-}\;+\;H_{2}$$

where M is the active site and *H is intermediate during HER. Experimental and theoretical efforts suggest that both advantageous water dissociation and ideal *H adsorption free energy (ΔG_*H_ = 0) are needed to guarantee a high catalytic HER activity in alkaline condition.^[^ ^77^, ^78^ ^]^

For water oxidation reaction (oxygen evolution reaction, OER) at the photoanode, proposed mechanism is (Figure 3c)

(5) $$M\;+\;OH^{-}\;\to \;M\;-\;*\mathrm{OH}\;+\;e^{-}$$

(6) $$M\;-\;*\mathrm{OH}\;+\;OH^{-}\;\to \;M\;-\;*O\;+\;H_{2}O_{\left(l\right)}\;+\;e^{-}$$

(7) $$2M\;-\;*O\;\to \;2M\;+\;O_{2\left(g\right)}\;$$

or

(8) $$M\;+\;OH^{-}\;\to \;M\;-\;*\mathrm{OH}\;+\;e^{-}$$

(9) $$M\;-\;*\mathrm{OH}\;+\;OH^{-}\;\to \;M\;-\;*O\;+\;H_{2}O_{\left(l\right)}\;+\;e^{-}$$

(10) $$M\;-\;*O\;+\;OH^{-}\;\to \;M\;-\;*\mathrm{OOH}\;+\;e^{-}$$

(11) $$M\;-\;*\mathrm{OOH}\;+\;OH^{-}\;\to \;M\;+\;O_{2\left(g\right)}\;+\;H_{2}O_{\left(l\right)}\;+\;e^{-}$$

where M is the active site, *OH, *O, and *OOH are the intermediates during OER. Notably, the mechanism of OER is very complex and involving four electrons. Theoretical calculations predict that the rate‐determining step of OER is the formation of *OOH (Equation (10)),^[^ ^79^ ^]^ and catalyst design should be guided to stabilize *OOH and its transition state on the surface of catalysts.

### 2.2 Mechanism for NRR

More than 150 million tons of NH_3_ is produced by Haber–Bosch process each year, accounting for 3–5% of the world's natural gas consumption, 1–2% of the world's annual energy supply, and ≈1.5% of global greenhouse gas emission.^[^ ^80^, ^81^, ^82^ ^]^ By contrast, PEC‐NRR is a very attractive route to produce NH_3_ at ambient temperature and atmospheric pressure.^[^ ^83^, ^84^, ^85^ ^]^

**Figure 4:**
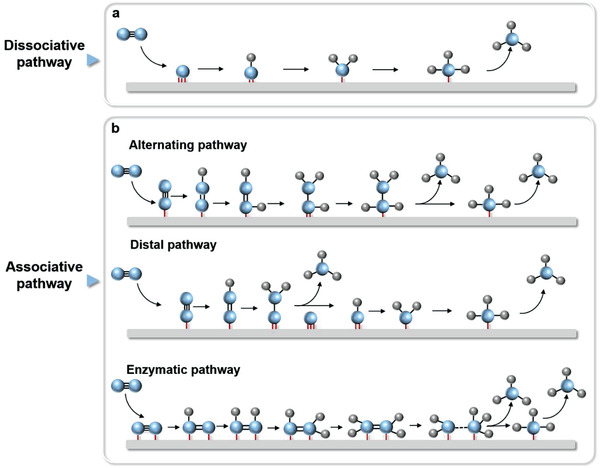
The possible reaction mechanisms of PEC‐NRR including a) dissociative and b) associative pathways.

Generally, a typical NRR process involves the N_2_ adsorption/activation and cleavage of the N≡N bond, the hydrogenation of intermediates and the final desorption of NH_3_.^[^ ^86^, ^87^ ^]^ There are two widely accepted mechanisms for NRR,^[^ ^88^, ^89^ ^]^ i.e., dissociation pathway (**Figure** **4**a) and association pathway (Figure 4b). In the dissociation pathway, N≡N triple bond in adsorbed N_2_ cleaves first, and then the two isolated N atoms adsorbed on the catalyst surface are separately hydrogenated to form two NH_3_ molecules. Due to the ultrastable N≡N triple bond, the dissociative mechanism requires extremely high energy. According to the different N_2_ adsorption modes and hydrogenation sequences,^[^ ^90^ ^]^ the association mechanism can be further divided into three pathways, namely alternating, distal, and enzymatic pathways (Figure 4b). In the alternating pathway, N_2_ molecules are adsorbed on the catalyst surface in the end‐on mode, and two N atoms are hydrogenated alternately to release two NH_3_ molecules. This process may produce by‐products (such as N_2_H_2_ and N_2_H_4_), thus reducing the Faradaic efficiency and yield of NH_3_.^[^ ^91^, ^92^ ^]^ In the distal pathway, hydrogenation preferentially occurs on the distal N atom of the N_2_ molecule, first releasing an NH_3_ molecule and leaving another N atom on the surface to generate another NH_3_.^[^ ^90^, ^93^ ^]^ For the enzymatic pathway, N_2_ molecules are adsorbed on the catalyst surface in the side‐on mode, and the hydrogenation process is similar to that in alternating mode.

Notably, the well‐known Haber–Bosch method proceeds in the dissociation pathway, which requires considerable energy to break the ultrastable N≡N triple bond (940.95 kJ mol^−1^).^[^ ^94^, ^95^, ^96^ ^]^ This is the origin of the high energy consumption of the Haber–Bosch method. By contrast, most of catalysts for sustainable NH_3_ production such as noble metal, non‐noble metal, or nonmetal, are reported to catalyze NRR in the association mechanism.^[^ ^97^, ^98^ ^]^

### 2.3 Mechanism for CRR

PEC reduction of CO_2_ is a promising route to convert CO_2_ into valuable chemical products such as CO, CH_4_, CH_3_OH, C_2_H_4_, C_2_H_5_OH, etc.^[^ ^99^ ^]^ CRR process is very complex, and the products are diverse, which are highly dependent on the reaction routes. The widely acknowledged pathways include C_1_ and C_2_.^[^ ^100^ ^]^

**Figure 5:**
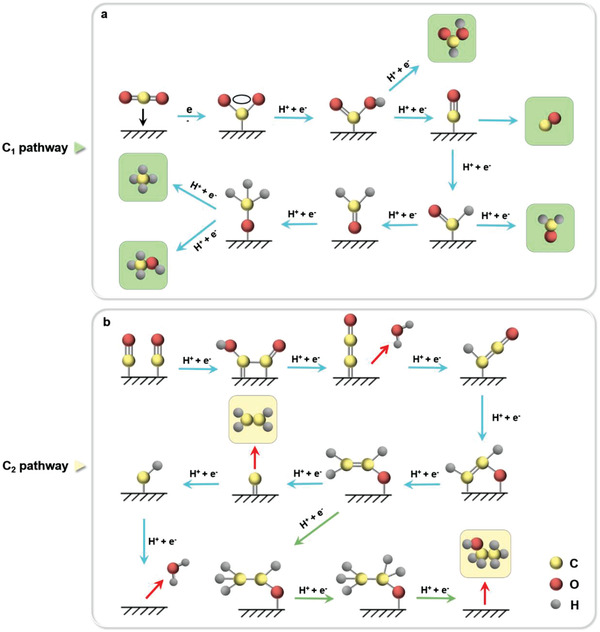
The possible reaction pathways of different CO_2_ reduction products: a) C_1_ pathway; b) C_2_ pathway.

For the C_1_ pathway (**Figure** **5**a), the first electron reduces CO_2_ into *CO_2_^−^, and the second proton–electron pair transferring to the C or O atom in *CO_2_^−^ yields HCOOH or CO, respectively. The further proton–electron pairs can reduce the adsorbed CO (CO*) to CH_4_ or CH_3_OH.^[^ ^101^ ^]^ Compared with the straightforward C_1_ reaction routes, the reaction routes for C_2_ products are more complex. To date, there are still some disputes about the possible pathways for forming C_2_ products,^[^ ^102^, ^103^ ^]^ such as *CO dimerization, *CO—COH coupling, “carbene,” CO insertion mechanism, etc. In the path from *CO to C_2_H_4_ or CH_3_CH_2_OH, the formation of *CO—COH is considered as the rate‐determining step (Figure 5b).^[^ ^39^, ^104^ ^]^ Then, *CO—COH is reduced to *CH_2_CHO via several proton–electron coupled transfer steps. And *CH_2_CHO is subsequently transformed to ethylene or ethanol via hydrogenolysis or hydrogenation processes.^[^ ^39^ ^]^

Although possible reaction mechanisms of water splitting (OER and HER), NRR, and CRR have been proposed, revealing the detailed mechanism still remains a major challenge because of the short lifetime and low coverage of intermediates under working conditions. Combining cutting‐edge in situ characterization techniques with advanced theoretical calculations opens up new exciting opportunities to reveal the “real” catalytic reactions.

## 3 Advancing PEC Reactions through Atomic Design of Photoelectrodes

**Figure 6:**
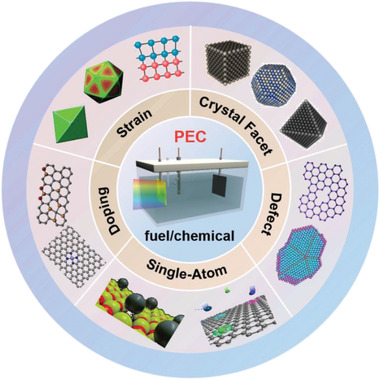
Schematic diagram of enhancing PEC‐driven solar‐to‐fuel/chemical conversions by the atomic design of photoelectrocatalysts.

As mentioned above, the photo‐to‐energy (fuel or chemical) conversion efficiency of the PEC system is closely related to the mechanisms of OER, HER, NRR, and CRR occurring at the photoelectrocatalysts. Recent experimental and theoretical works^[^ ^105^, ^106^, ^107^ ^]^ demonstrate that the catalytic mechanisms (thermodynamic barrier,^[^ ^108^ ^]^ kinetic barrier,^[^ ^109^ ^]^ rate‐determining step,^[^ ^110^ ^]^ etc.) of the PEC‐driven catalytic reactions are very sensitive to electrode surface structure. And materials with different compositions/structures exhibit quite different reactivity. Therefore, adjusting the atomic structure of photoelectrocatalysts by creating defects, strain (**Figure** **6**), dopants, or single atoms is an effective way to improve the performance of PEC devices.

### 3.1 Defect Engineering

**Figure 7:**
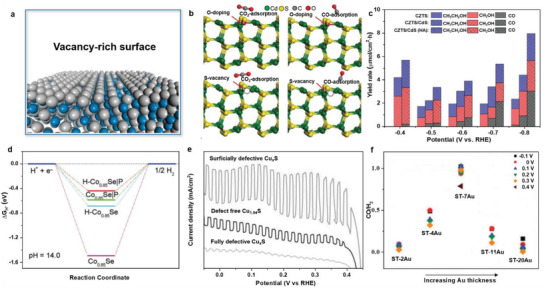
a) Schematic diagram of surface vacancy defects. b) DFT‐optimized structures of CO_2_ and CO adsorbed on CdS and O‐doped CdS with S‐vacancy. c) Yield rates of CO_2_ reduction products under illumination. Reproduced with permission.^[^ ^116^ ^]^ Copyright 2021, Wiley‐VCH. d) DFT‐calculated HER free‐energy diagrams. ^[^ ^117^ ^]^ Copyright 2021, Wiley‐VCH. e) Linear sweep voltammetry curves of different Cu_1.94_S nanoflake arrays under the chopped full‐spectrum illumination. Reproduced with permission.^[^ ^119^ ^]^ Copyright 2017, Wiley‐VCH. f) CO to H_2_ ratios at different potentials on various amorphous‐Si/TiO_2_/Au electrodes. Reproduced with permission.^[^ ^124^ ^]^ Copyright 2019, The Royal Society of Chemistry.

Defects (**Figure** **7**a) can modulate the local electronic structure of the catalysts, facilitate the rate‐determining step, reduce the reaction energy barrier, and sometimes become active sites themselves for PEC‐driven energy conversion.^[^ ^111^, ^112^, ^113^, ^114^ ^]^

Recently, Shi and co‐workers fabricated O‐vacancy‐rich BiOI nanosheets for PEC‐NRR.^[^ ^115^ ^]^ They demonstrated that the O‐vacancies significantly enhanced the adsorption of N_2_ molecules on BiOI nanosheets, evidenced by a temperature programmed desorption experiment. Moreover, they claimed that O‐vacancies could effectively inject the photogenerated electrons into the antibonding orbital of the adsorbed N_2_ molecule, thus weakening the N≡N bond and promoting N_2_ reduction.

Hao and co‐workers reported the control of the selectivity of PEC‐CRR by adjusting the S‐vacancy on the surface of Cu_2_ZnSnS_4_/CdS photocathode by a facile heat treatment in different atmospheres (air and nitrogen).^[^ ^116^ ^]^ They revealed that after heat treatment in air, the S‐vacancies on the Cu_2_ZnSnS_4_/CdS surface were filled by oxygen, leading to better CO_2_ and CO adsorption capability with enhanced CRR activity and higher selectivity toward methanol/ethanol (Figure 7b). After heat treatment in N_2_, more generated S‐vacancies facilitated the surficial CO desorption process and higher CO selectivity (Figure 7c).

Feng and co‐workers reported porous cobalt phosphoselenide nanosheets with Se‐vacancies for highly efficient PEC water splitting.^[^ ^117^ ^]^ Their density functional theory (DFT) calculations revealed that the Se‐vacancies and P atoms synergistically regulated the electronic structure of the catalysts, thus optimized the ΔG_*H_ and promoting HER kinetics (Figure 7d). As a result, their PEC water‐splitting device using the cobalt phosphoselenide nanosheet as both the anode and cathode achieved a hydrogen generation current density of 10 mA cm^−2^ at a low voltage of 1.64 V, which exceeded the Ir/C–Pt/C reference device.

Recently, engineering dual‐/multivacancy defects demonstrated the great potential to enhance the PEC performance of photoelectrodes.^[^ ^118^ ^]^ Wang et al. developed a multistep heat treatment strategy to synthesize a g‐C_3_N_4_/reduced graphene oxide (RGO)/TiO_2_ composite with both N‐ and O‐vacancies. They showed that benefiting from the extended visible‐light absorption range and the enhanced carrier separation/transfer rate arising from the defects, the composite exhibited a greatly enhanced photocatalytic H_2_ evolution rate of 4760 µmol h^−1^ g^−1^, surpassing most reported g‐C_3_N_4_/TiO_2_ composites.

In addition to anion vacancies, Du and co‐workers reported manipulating Cu‐vacancies in Cu_2‐_ *_x_* S nanoflake arrays to modulate the PEC‐HER performance.^[^ ^119^ ^]^ They showed that the Cu_2‐*x*_S nanoflake arrays with surface‐rich Cu‐vacancies exhibited a considerably enhanced PEC‐HER performance (Figure 7e). They claimed that such superior performance originates from the Cu‐vacancies optimized surface catalytic property, which facilitated charge separation and resulted in surface plasmon resonance effect as well.

Besides point defects, such as vacancies described above, planar defects^[^ ^120^, ^121^, ^122^, ^123^ ^]^ were employed to regulate the PEC performance of catalysts. Gong and co‐workers reported grain‐boundary‐rich gold‐catalyst‐decorated amorphous silicon photocathode to produce syngas with adjustable CO/H_2_ ratios (Figure 7f).^[^ ^124^ ^]^ They demonstrated that CO selectivity was strongly associated with the grain‐boundary density of the Au catalysts. They showed that the grain boundary induced lattice strain at the surface, which effectively stabilized the *COOH intermediate, thus enhancing the CRR activity. As a result, the designed system achieved a half‐cell efficiency of 0.42% for CRR under 1 sun illumination. Moreover, Maschmeyer and co‐workers synthesized high‐index faceted rutile TiO_2_ thin films with twin grain boundaries for efficient PEC water splitting.^[^ ^125^ ^]^ They demonstrated that the twin boundaries improved the interaction at the TiO_2_/electrolyte interface, thus enhancing the catalytic activity of the PEC device.

Despite substantial progress in enhancing PEC performance through introducing defects, there are still many unsolved issues. For example, precise control of the concentration and distribution of defects is highly required,^[^ ^126^ ^]^ because the effects of defect concentrations, distribution, and interaction between different types of defects on catalyst activity are different. Besides, in photoelectrocatalysts, the presence of large quantities of defects usually sacrifices the efficiency in separation and transport of photogenerated electrons/holes, so the defects should be located at the outermost surface of photoelectrocatalysts to just facilitate the catalytic reactions. In synthesis, the post‐treatment^[^ ^127^ ^]^ or heat treatment^[^ ^128^ ^]^ of presynthesized catalysts is more conducive to the distribution of defects on the outermost surface of the catalysts. More importantly, compared with point defects, planar defects can provide more active sites in catalysts.^[^ ^129^, ^130^ ^]^ However, planar defects are not stable in small‐sized catalysts, so it is still a great challenge to construct well‐controlled planar defects in catalysts.

### 3.2 Heteroatom Doping

**Figure 8:**
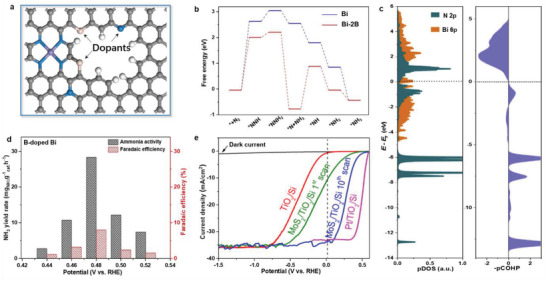
a) Schematic diagram of heteroatom doping. b) DFT‐calculated NRR free‐energy diagrams on pristine and B‐doped Bi catalysts. c) Projected density of states (pDOS) and projected crystal orbital Hamilton populations (pCOHP) for the N atoms of *NNH directly bonded to Bi atom on optimum B‐doped Bi catalysts. d) NH_3_ yield rate and Faradaic efficiency of B‐doped Bi nanorolls. Reproduced with permission.^[^ ^142^ ^]^ Copyright 2020, Elsevier B.V. e) Linear sweep voltammetry curves of PEC‐HER photocathodes. Reproduced with permission.^[^ ^143^ ^]^ Copyright 2020, American Chemical Society.

Heteroatom doping can significantly change the physical and chemical properties of materials,^[^ ^131^, ^132^, ^133^, ^134^ ^]^ such as 1 ppm boron (B) doping can alter the resistivity of silicon by five orders of magnitude. In electrocatalysis, heteroatom doped carbon‐based metal‐free materials are widely used for oxygen reduction reaction (ORR),^[^ ^135^ ^]^ OER,^[^ ^136^ ^]^ HER,^[^ ^137^ ^]^ and NRR.^[^ ^138^, ^139^ ^]^ Recently, heteroatom doping (**Figure** **8**a) has shown great potential in PEC‐driven catalytic reactions.^[^ ^140^, ^141^ ^]^

For example, Xiong and co‐workers incorporated B in Bi catalysts for enhanced PEC‐NRR.^[^ ^142^ ^]^ Note that the first hydrogenation step of N_2_ to form *NNH is the rate‐determining step for NRR. Their theoretical calculation revealed that, in the case of B‐doped Bi catalyst, the energy barrier of *NNH formation was reduced to 2.0 eV, much lower than the pristine catalyst (Figure 8b). Meanwhile, the B dopants could activate nearby Bi atoms as active sites for NRR and donate electrons to Bi, bringing more electrons into the antibonding orbital of *NNH (Figure 8c). As a result, their PEC system achieved an ammonia yield rate of 29.2 mg NH_3_ g_cat._^−1^ h^−1^ and Faradaic efficiency of 8.3% at a bias of 0.48 V versus reversible hydrogen electrode (RHE) (Figure 8d).

Shen and co‐workers reported the activation of the MoS_2_ basal plane for PEC‐HER by in situ O‐doping.^[^ ^143^ ^]^ Based on combined experimental results and theoretical calculations, they demonstrated that the O—Mo—S sites on the MoS_2_ basal planes enhanced the intrinsic conductivity of MoS_2_ by strengthening the hybridization between Mo‐d orbital and S‐p orbital. Moreover, the O‐doping effectively modulated ΔG_*H_ on the MoS_2_ basal plane. This significantly promoted the PEC‐HER activity of the MoS_2_ basal plane (Figure 8e).

Liu and co‐workers reported a P‐doped MoS_2_ as a HER cocatalyst for p‐Si electrode.^[^ ^144^ ^]^ Their extended X‐ray absorption fine structure spectra and DFT calculations indicated that the doped P atoms created more exposed edges and S‐vacancies. In particular, S‐vacancies activated the catalytic activity of the inert basal plane. Moreover, Bemana and Nadimi successfully doped S atoms into the hematite matrix,^[^ ^145^ ^]^ and found that the incorporated S atoms were more probable in the form of cationic substitution (S^4+^) rather than anionic substitution (S^2−^). This resulted in more O‐vacancies and Fe sites with adjustable valence state, which improved the mobility of the photoinduced charge carrier, thus favoring the PEC performance.

Generally, heterodopants are incorporated into the host PEC materials during the growth of the host materials in traditional chemical approaches^[^ ^146^, ^147^, ^148^, ^149^ ^]^ or post‐treatment of the presynthesized host materials.^[^ ^150^ ^]^ However, heteroatoms are often repelled out of the host material due to the “self‐purification” effect,^[^ ^54^ ^]^ which results in poor dopant stoichiometric control. Similar to the defect engineering discussed above, precise control of the concentration and distribution of heterogeneous dopants on the surface of electrocatalysts still remains challenging.

### 3.3 Facet Engineering

**Figure 9:**
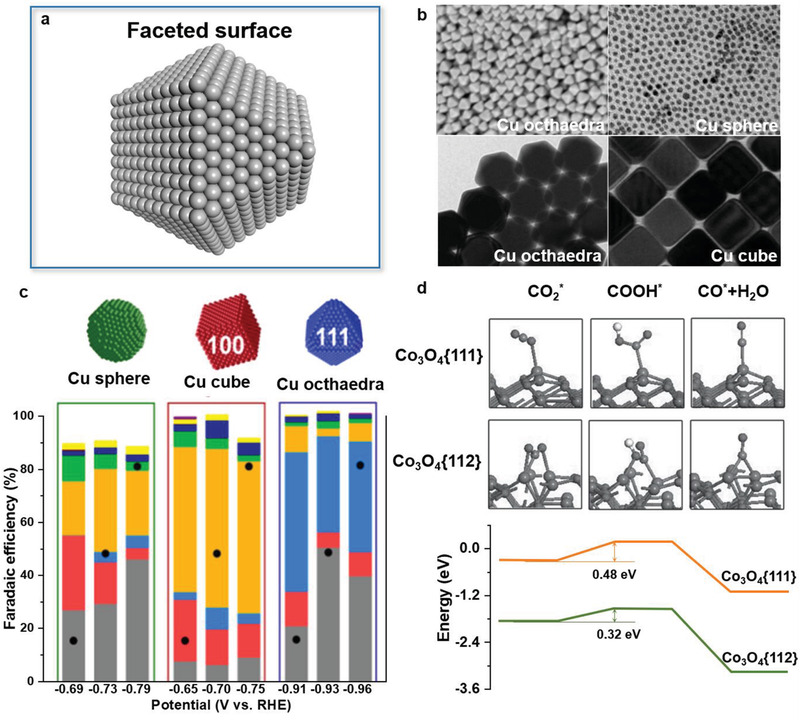
a) Schematic diagram of faceted catalyst surface. b) Scanning electron microscopy (SEM) and transmission electron microscopy (TEM) images of various faceted Cu nanocrystals. c) Faradaic efficiencies of Cu catalysts with spherical, cubic, and octahedral morphologies in a gas‐fed flow cell in 1 m KOH. Reproduced with permission.^[^ ^163^ ^]^ Copyright 2020, American Chemical Society. d) DFT‐optimized structures and free energy diagram of CO_2_ and reduction intermediates on Co_3_O_4_{111} and Co_3_O_4_{112} surfaces. Reproduced with permission.^[^ ^164^ ^]^ Copyright 2016, WILEY‐VCH.

Due to the different adsorption/desorption characteristics of reactants and reaction intermediates on the various exposed facets of catalysts, facet‐dependent catalysis has been widely observed in thermocatalysis, photocatalysis, and electrocatalysis.^[^ ^151^, ^152^, ^153^, ^154^ ^]^ Undoubtedly, controlling the exposure of crystal facets (**Figure** **9**a) on the PEC material is one of the most effective ways to improve the PEC activity.^[^ ^155^ ^]^

Water interaction with the photoelectrode surface is crucial for PEC water splitting. H_2_O adsorption on TiO_2_ surfaces has been thoroughly investigated.^[^ ^156^, ^157^, ^158^ ^]^ Theoretical and experimental studies have revealed that unsaturated Ti and O atoms on the surface of TiO_2_ adsorb water molecules by forming Ti—O and O—H bonds, respectively. Apparently, surface facets with varied densities of unsaturated Ti and O atoms exhibit significantly different water adsorption and dissociation characteristics. Besides TiO_2_, WO_3_ is also widely used as photoelectrocatalyst for PEC water splitting.^[^ ^159^, ^160^ ^]^ Wang and co‐workers fabricated a WO_3_ film with preferentially exposed {002} facets.^[^ ^161^ ^]^ They showed that the PEC performance of this film was much better than that of WO_3_ nanoplate arrays exposing {200} and {002} facets. In particular, WO_3_ film with the dominant {002} facet exhibited a high photocurrent density of 3.7 mA cm^−2^ at 1.23 V versus RHE, ≈93% of the theoretical photocurrent of WO_3_. Their theoretical calculations revealed that the overall energy barrier to achieve water oxidation reaction was lower on the {002} facet than that on the {200} facet. Moreover, the excellent PEC performance of the WO_3_{002} facet was observed by the work of Gong and co‐workers.^[^ ^162^ ^]^

In addition to PEC water splitting, Buonsanti and co‐workers reported the facet‐dependent activity and selectivity of differently shaped Cu nanoparticles in a gas‐fed flow cell configuration (Figure 9b).^[^ ^163^ ^]^ Their experimental results showed that Cu cubes with exposed {100} facets increased ethylene selectivity up to 57%, and Cu octahedra with exposed {111} facets achieved a methane selectivity up to 51% (Figure 9c). In addition, Gao et al. prepared Co_3_O_4_ hexagonal platelets with mainly exposed {112} crystal facets for CRR.^[^ ^164^ ^]^ The combination of experiment and calculation disclosed that the exposed {112} facets of Co_3_O_4_ hexagonal platelets were essential for capturing and activating CO_2_ molecules (Figure 9d). Specifically, the proton transfer step was greatly facilitated on the {112} facets of Co_3_O_4_, which is generally considered as the rate‐determining step for the overall CRR.

The above work shows that controlling the exposed facets of photoelectrocatalysts is an effective way to improve the performance. At present, the exposed facets of noble metal nanocatalysts can be realized by controlling the crystal morphology, such as icosahedron,^[^ ^165^, ^166^ ^]^ dodecahedra,^[^ ^167^, ^168^ ^]^ decahedron,^[^ ^169^, ^170^, ^171^ ^]^ tetrahedron,^[^ ^172^, ^173^ ^]^ cube,^[^ ^174^ ^]^ etc. However, the accurate regulation of the exposed facets on most non‐noble metal catalysts is still a big challenge. Moreover, the more active facets are also prone to change in the PEC catalytic reactions. Therefore, special attention should be paid to the stability of the exposed crystal facets in working conditions and the influence of crystal facets on catalytic activity.

### 3.4 Strain Engineering

**Figure 10:**
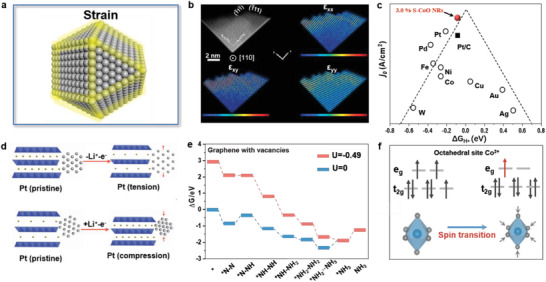
a) Schematic illustration of lattice strain. b) Contour plots of the strain components on a CoO catalyst. c) HER volcano plot. Reproduced with permission.^[^ ^181^ ^]^ Copyright 2017, Springer Nature. d) Schematic of the lattice change of LiCoO_2_. Reproduced with permission.^[^ ^182^ ^]^ Copyright 2016, American Association for the Advancement of Science. e) NRR free‐energy diagrams of graphene. Reproduced with permission.^[^ ^183^ ^]^ Copyright 2021, Elsevier B.V. f) Schematic diagram of Co^2+^ electronic configurations in high‐ and low‐spin states. Reproduced with permission.^[^ ^185^ ^]^ Copyright 2020, Wiley‐VCH.

Strain engineering (**Figure** **10**a), by expanding or compressing atoms in a crystal lattice, has recently proven to be a powerful strategy altering the electronic structure and reactivity of materials.^[^ ^175^, ^176^, ^177^, ^178^, ^179^ ^]^ For example, the exceptionally active Pt_3_Ni ORR catalysts were obtained by adopting Ni atoms in the underlying atomic layers to compress the Pt surface layer, resulting in a significant d‑band center downward shift of the Pt surface, thus leading to optimum adsorption of oxygenated intermediate species and ultrahigh ORR activities.^[^ ^180^ ^]^

We have reported boosting the HER performance of CoO nanorods by engineering surface strain.^[^ ^181^ ^]^ Notably, transition metal oxides are generally known to be inactive toward HER due to their inappropriate ΔG_*H_ (too strong H* adsorption on O, but too weak on metal ion). Our experimental and theoretical work showed that the tensile strain on the surface of CoO nanorods significantly reduced the O‐vacancy formation energy, thus promoting the formation of massive O‐vacancies as the active sites for water dissociation (Figure 10b). Moreover, we found that the tensile strain can tune the electronic structure of CoO nanorods to achieve the optimum ΔG_*H_. Moreover, we emphasized that the intrinsic activity of the tensile strain engineered CoO nanorods was located at the top of the HER volcano plot (Figure 10c).

Cui and co‐workers used an electrode material in lithium‐ion battery, LiCoO_2_, as the medium to control the lattice strain of the loaded Pt catalyst.^[^ ^182^ ^]^ They showed that the volume and lattice spacing of layered LiCoO_2_ could be changed by the chemical extraction/intercalation of Li ions in the electrochemical charge/discharge process (Figure 10d). This resulted in the compressive or tensile strain in Pt catalyst, which could effectively regulate the adsorption binding energy of intermediates, and thus the catalytic ORR activity of Pt catalysts. They observed a 90% enhancement in Pt ORR activity under compressive strain.

Mao and co‐workers investigated the effect of strain on the catalytic NRR performance of graphene by DFT calculations.^[^ ^183^ ^]^ They found that the increase in strain on graphene led to a decrease in the energy barrier of NRR (Figure 10e), and the graphene with a strain magnitude of 8% exhibited an ultralow energy barrier of 0.35 eV and an overpotential of 0.19 V.

In addition, the enlargement or contraction of the crystal lattice can affect the spin state of magnetic catalysts, which plays a key role in determining their catalytic activity.^[^ ^184^ ^]^ Our computational results demonstrated that the contraction of Co—O octahedral in Co_2_VO_4_ strengthened the crystal field and increased the energy gap between t_2g_ and e_g_, thus causing the spin transition of Co ions from the high‐spin state (t_2g_^5^e_g_^2^) to the low‐spin state (t_2g_^6^e_g_^1^). This is highly advantageous for ORR and OER (Figure 10f).^[^ ^185^ ^]^

Although the works mentioned above are mainly in the field of electrocatalysis, we believe that strain engineering may provide a new approach for the development of high‐performance PEC electrodes. We note that lattice strain, including compression or tension, is usually generated through lattice mismatch between materials, which requires the construction of core–shell structures or the introduction of substrate materials.^[^ ^179^ ^]^ This undoubtedly poses a challenge to the design of PEC electrodes, because the PEC electrode also undertakes the functions of light absorption, charge separation, and transfer at the same time. One possible solution to introduce lattice strain only on the surface of PEC electrode is through facet engineering or incorporating defects or dopants,^[^ ^181^ ^]^ which will not affect the light absorption, charge separation, and transfer in the bulk electrode.

### 3.5 Single‐Atom Engineering

**Figure 11:**
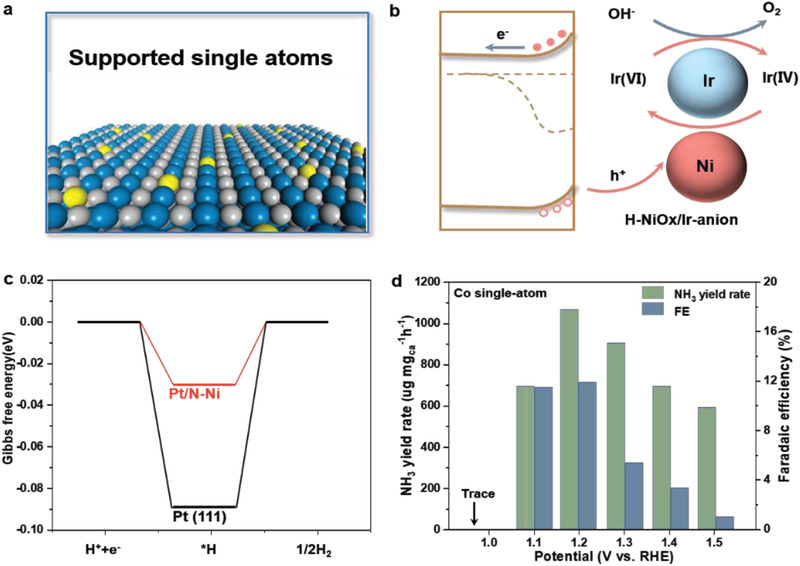
a) Schematic diagram of single atoms supported on the surface of a catalyst. b) Proposed OER reaction paths for Fe_2_O_3_–NiO*_x_*–Ir single‐atom catalyst. Reproduced with permission.^[^ ^192^ ^]^ Copyright 2017, Springer Nature. c) HER free energy diagram on the Pt(111) surface and single‐atom Pt anchored on Ni surface, referred to as Pt_1_/N—Ni. Reproduced with permission.^[^ ^190^ ^]^ Copyright 2021, The Royal Society of Chemistry. d) PEC‐NRR performance of cobalt single‐atom catalyst on CoPi/Ti–Fe_2_O_3_ photoanode. Reproduced with permission.^[^ ^193^ ^]^ Copyright 2021, Elsevier B.V.

Single‐atom catalysts (**Figure** **11**a) have become one of the frontiers and hot topics in the fields of materials, chemistry, and physics.^[^ ^186^, ^187^, ^188^, ^189^ ^]^ Recently, single‐atom catalysts have shown promising applications in PEC devices.^[^ ^190^, ^191^ ^]^

Cui et al. developed a Fe_2_O_3_–NiO*_x_*–Ir single‐atom electrode for PEC‐OER,^[^ ^192^ ^]^ in which NiO*_x_* acted as the “movable bridge” connecting Fe_2_O_3_ with Ir single atom, capturing the photogenerated holes from Fe_2_O_3_ and transferring them to Ir single atom; while Ir single atom served as the super active sites for OER (Figure 11b). As a result, they observed an unusual OER activity with a turnover frequency of 2.4‐12.7 s^−1^ per Ir site at 1.23 V versus RHE and a high stability of the hybrid catalyst (>80 h) in alkaline electrolyte.

Very recently, Zhang and co‐workers dispersed Pt single atoms on the nitrogen modified Ni substrate (Pt_1_/N—Ni) for PEC‐HER.^[^ ^190^ ^]^ They demonstrated that the lone pair electrons in nitrogen atoms were prone to coordinate with the unoccupied d orbital of Pt atoms, stabilizing Pt single atoms. This was supported by the clustering energy calculation (Figure 11c). They further integrated the Pt_1_/N—Ni catalyst into the Cu_2_O nanowire photocathodes for PEC‐HER. The hybrid electrode achieved an extremely high photocurrent density of 11.9 mA cm^−2^ at 0 V versus RHE and a high photo‐to‐H_2_ conversion efficiency of 1.75%.

Zhao and co‐workers reported an efficient PEC‐NRR system by integrating the Fe_2_O_3_‐based photoanode for water oxidation and the Co single‐atom‐based photocathode for NRR.^[^ ^193^ ^]^ This system afforded an NH_3_ generation rate of 1021.5 µg mg_Co_^−1^ h^−1^ and a Faradaic efficiency of 11.9% at 1.2 V versus RHE (Figure 11d).

In the above works, the deposited single atoms on the photoelectrodes act as the active sites for PEC reactions. Apparently, the low loading mass of single atoms on the photoelectrodes greatly limits the photo‐to‐energy (fuel/chemical) conversion of the PEC system. Therefore, it is highly required to develop a synthetic approach for high loading of single atoms. Very recently, a graphene‐quantumdot‐mediated method has promoted the single‐atom loading up to 40 wt%,^[^ ^194^ ^]^ which provides a new direction in this field. On the other hand, single‐atom‐modified photoelectrodes are also expected to find wide applications for reactions beyond HER and OER, such as NRR and CRR.

## 4 Conclusions and Future Perspectives

This review summarizes the recent advances in the atomic design of photoelectrocatalysts toward HER, NRR, and CRR from the perspective of surface catalytic reactions. We introduce basic catalytic mechanisms and show the commonly used strategies for the design of efficient photoelectrocatalysts at the atom scale, including engineering of defect, dopant, facet, strain, and single atom. Although some achievements have been made, surface catalytic reactions have not been paid adequate attention to PEC systems, and most research works are focusing on improving light absorption and charge separation/transfer. Further research efforts should be devoted to filling this gap in order to improve the performance of PEC. The ultimate goal is to understand the PEC reactions at the molecular level and design PEC materials atom‐by‐atom to boost the catalytic activity and selectivity. Future research direction is suggested but is not limited to:

1. At present, the understanding of catalytic mechanisms of HER, NRR, and CRR reactions in the PEC system is mainly inherited from the corresponding electrocatalytic reactions. But compared with electrocatalytic reactions, PEC catalytic reactions are distinct in some aspects. For example, a large amount of hot electrons are generated under light illumination, which can significantly change the catalytic reaction mechanisms. More experimental and theoretical efforts should be devoted to revealing the “real” PEC catalytic reaction mechanisms.
2. Recently, rapid progress in *insitu* surface‐enhanced spectroscopies, such as infrared absorption,^[^ ^195^, ^196^ ^]^ Raman,^[^ ^197^, ^198^ ^]^ and advanced theoretical calculations^[^ ^199^, ^200^ ^]^ opens up new exciting opportunities for monitoring the reactive intermediates with short lifetime and low coverage under operando conditions, which have achieved great success in thermocatalysis^[^ ^201^ ^]^ and electrocatalysis.^[^ ^202^ ^]^ Extending these advanced technologies and successful research experiences to PEC systems will undoubtedly bring new breakthroughs in PEC‐driven photo‐to‐energy conversions. The main challenge now is to design optical paths to eliminate the interference of the incident light on the Raman or infrared signals in these cutting‐edge in situ spectroscopies.
3. PEC‐NRR and PEC‐CRR are still in their infancy, whose activity and selectivity are far from satisfactory for industrial application. In PEC‐NRR and PEC‐CRR, PEC‐HER is a competing reaction with favorable thermodynamics and kinetics, which largely limits the production rate and selectivity of the desired products in PEC‐NRR and PEC‐CRR. Recently, taking advantage of the unique localized surface plasmon resonance,^[^ ^203^, ^204^ ^]^ photothermal effect in the PEC system^[^ ^205^, ^206^ ^]^ to activate the reactants and intermediates has been proved a highly promising way to decrease the reaction barrier, facilitate the rate‐determining step, and thereby promote the overall catalytic activity and selectivity of PEC‐NRR and PEC‐CRR. Therefore, more attentions should be paid to these unique light‐induced effects.
4. Exploring a new PEC catalyst paradigm with an efficient solar energy utilization in the untapped near‐infrared range can promote the development of full‐spectrum‐driven PEC systems. In this field, metal‐assisted or self‐doped plasmonic semiconductors have shown great potential due to their strong absorption in the near‐infrared range.

## Conflict of Interest

The authors declare no conflict of interest.
